# Risk factors for community-onset urinary tract infections caused by extended-spectrum β-lactamase-producing Escherichia coli*

**DOI:** 10.3906/sag-1902-24

**Published:** 2019-08-08

**Authors:** Türkan TÜZÜN, Selda SAYIN KUTLU, Murat KUTLU, Ilknur KALELİ

**Affiliations:** 1 Department of Infectious Diseases and Clinical Microbiology, Denizli Surgery Hospital, Denizli Turkey; 2 Department of Infectious Diseases and Clinical Microbiology, Faculty of Medicine, Pamukkale University, Denizli Turkey; 3 Department of Medical Microbiology, Faculty of Medicine, Pamukkale University, Denizli Turkey

**Keywords:** Community-onset, epidemiology, extended-spectrum β-lactamase, risk factors, urinary tract infections

## Abstract

**Background/aim:**

Community-onset urinary tract infections (UTIs) caused by extended-spectrum β-lactamase (ESBL)-producing *Escherichia coli* have increased in many parts of the world. This study aimed to determine the prevalence and risk factors for community-onset UTI caused by ESBL-producing *E. coli*.

**Materials and methods:**

This prospective cohort study was conducted between January 2012 and March 2014 in cases of community-onset UTI caused by *E. coli*. Patients with UTI due to ESBL-producing *E. coli* and patients with UTI due to non-ESBL-producing *E. coli* were compared to identify risk factors for ESBL-producing *E. coli *in the community.

**Results:**

A total of 305 patients (116 males [46.4%]; mean age: 57.76 ± 18.06 years) were included in the study. Among these patients, 154 (50.5%) were infected with ESBL-producing *E. coli*. In multivariate analysis, the healthcare-associated UTI (odds ratio [OR]: 1.80; 95% confidence interval [CI]: 1.02–3.18; P = 0.041), upper urinary tract infection (OR: 3.05; 95% CI: 1.76–5.29; P < 0.0001), use of antibiotics in the preceding 6 months (OR: 2.28; 95% CI: 1.21–4.30; P = 0.011), and having two or more risk factors (OR: 4.03; 95% CI: 1.73–9.35; P = 0.001) were the significant factors associated with increased risk of community-onset UTIs due to ESBL-producing *E. coli*.

**Conclusion:**

The increasing prevalence of**ESBL-producing *E. coli* makes it difficult to decide the empirical therapy in UTIs, especially in patients with two or more of the risk factors. A better understanding of the epidemiology and risk factors associated with community-onset UTIs due to ESBL-producing *E. coli* may have significant implications in decision-making for empirical antimicrobial treatment.

## 1. Introduction

Urinary tract infections (UTIs) are the most common community-onset infections in the adult population in many parts of the world. Clinical manifestations of community-onset UTIs range from asymptomatic bacteriuria to acute pyelonephritis with sepsis [1,2]. The major UTI pathogen is *Escherichia coli*, and there is increasing multidrug resistance in the isolates from community-onset infections. Multidrug resistance of these isolates is frequently associated with the presence of extended-spectrum β-lactamase (ESBL) genes [3,4]. ESBL-producing isolates are resistant to all penicillins, cephalosporins, and aztreonam, combined with high resistance rates to fluoroquinolones and trimethoprim/sulfamethoxazole (TMP-SMX) [5]. Multidrug resistance of ESBL-positive *E. coli* makes it more difficult to decide the antibiotic treatment in community-onset UTI and increases the risk of treatment failure. Early initiation of appropriate empirical therapy reduces mortality, especially in life-threatening UTIs [6]. Therefore, a better understanding of the risk factors for community-onset UTIs caused by ESBL-positive *E. coli *will guide clinicians in choosing appropriate empirical therapy. Also, it will ensure that measures are taken to reduce risk factors for these resistant infections.

For this reason, we aimed to determine the prevalence and risk factors for community-onset UTI caused by ESBL-producing *E. coli*. 

## 2. Materials and methods 

### 2.1. Study population and data collection

This prospective cohort study was conducted between January 2012 and March 2014 in cases of community-onset UTI caused by *E. coli*. Demographic characteristics, underlying diseases, and clinical signs and symptoms of the patients, as well as microbiological data, were recorded. This study was conducted with the approval of the Medical Ethics Committee of Pamukkale University (date: 17 January 2012; resolution number: 02) and adhered to the principles of the Declaration of Helsinki. Individual informed consent was obtained from all study participants. 

Patients admitted with clinical signs and/or symptoms of UTIs (e.g., fever >38 °C, urgency, frequency, dysuria, or suprapubic tenderness) with no other recognized cause, pyuria (i.e. urine specimen with ≥10 white blood cells/mm3), and a positive urine culture were enrolled in the study [7]. 

UTI was defined as “community-onset” when the infection occurred among nonhospitalized patients or <48 h after hospitalization. Community-onset UTIs include community-acquired UTIs and healthcare-associated UTIs [7]. Therefore, we included healthcare-associated UTIs according to previously published criteria: hospitalization in an acute care hospital for two or more days in the previous 90 days; residence in a nursing home or a long-term care facility; received intravenous therapy at home or in a day hospital; hemodialysis treatment; intravenous chemotherapy 30 days before the infection; received wound care or specialized nursing care in the preceding 30 days; presence of long-term indwelling urethral catheters or an invasive urinary tract procedure in the previous 30 days [8].

### 2.2. Microbiological analysis

Urine specimens were obtained from either first clean-catch midstream urine or urinary catheters. The isolated bacteria were identified by conventional methods in the microbiology laboratory. Positive urine cultures were defined when the bacterial growth was ≥103 colony forming units (CFU)/mL [9]. ESBL determination was performed phenotypically with ceftazidime/ceftazidime clavulanate and cefotaxime/cefotaxime clavulanate disks, as recommended by the Clinical and Laboratory Standards Institute (CLSI) [10]. Antimicrobial susceptibility testing was performed using the disk diffusion method, according to the CLSI standards. 

Patients with UTI due to ESBL-producing *E. coli* and non-ESBL-producing *E. coli* were compared in terms of demographic characteristics, underlying diseases, and microbiological data to identify risk factors for ESBL-producing *E. coli *in the community.

### 2.3. Statistical analysis

Continuous variables were compared by independent samples t-test, and categorical variables were compared by chi-square test or Fisher’s exact test for association. Differences were considered statistically significant at P < 0.05. Statistical calculations were performed using SPSS 17.0 for Windows (SPSS Inc., Chicago, IL, USA). In order to analyze the effect of the number of risk factors on ESBL-producing *E. coli*, we separated the patients based on existing risk factors. Patients with no risk factor, one risk factor, and two or more risk factors were analyzed. 

Backward stepwise multiple logistic regression was performed to identify the risk factors for community-onset UTI caused by ESBL-producing *E. coli* as the dependent variable. However, we did not include the risk factors that were also in the definition of healthcare-associated UTIs in the multivariate analysis.

## 3. Results 

A total of 305 patients (116 males (46.4%); mean age: 57.76 ± 18.06 years; range: 16–87 years) were included in the study. Among these patients, 154 (50.5%) were infected with ESBL-producing *E. coli*. 

Being male, old age (≥55 years), hospitalization within 3 months, use of antibiotics within the previous 6 months, healthcare-associated UTI, upper UTI, frequent UTIs (>3 times/year), presence of indwelling urethral catheter, renal failure, and neoplasia were significant risk factors for ESBL-producing *E. coli* among community-onset UTI cases in univariate analysis (P < 0.05). Moreover, patients with two or more risk factors were more likely to have ESBL-producing *E. coli*. ESBL positivity was lower in patients with one risk factor or no risk factors (P < 0.05) (Table 1). Healthcare-associated UTI was seen in 142/305 (46.6%) patients; indwelling urethral catheter use was the most common reason, with a rate of 102/142 (71.8%) patients.

**Table 1:** Characteristics of community-onset UTI caused by ESBL-producing Escherichia coli.

	ESBL-positive E. coli, n=154 (%)	ESBL-negative E. coli, n=151 (%)	P-value
Sex, male, n = 116 (%)	73 (47.4)	43 (28.5)	0.01
Age ≥55 years	101 (65.6)	78 (51.7)	0.014
Hospitalization within 6 monthsLength of hospital stay, days, mean ± SD	73 (47.4)16.86 ± 18.07	22 (14.6)15.95 ± 15.71	<0.00010.83
Use of antibiotics within previous 6 monthsFluoroquinolonesCephalosporinsBeta-lactam-beta-lactamase inhibitors	62 (40.3)24 (16.0)28 (18.3)6 (3.9)	20 (13.2)11 (7.3)6 (4.0)2 (1.3)	<0.00010.019<0.0010.16
Healthcare-associated UTI	98 (63.6)	44 (29.1)	<0.0001
Upper UTI	94 (61.0)	36 (23.8)	<0.0001
Frequent UTIs (>3 times/year)	45 (29.2)	16 (10.6)	<0.0001
Indwelling urethral catheter	75 (48.7)	30 (19.9)	<0.0001
Diabetes mellitus	41 (26.6)	45 (29.8)	0.53
Renal failure	54 (35.1)	23 (15.2)	<0.0001
Nephrolithiasis	28 (18.2)	25 (16.6	0.708
Renal transplantation	6 (3.9)	2 (1.3)	0.160
Use of corticosteroid	11 (7.1)	10 (6.6)	0.858
Chronic obstructive lung disease	13 (8.4)	9 (6.0)	0.402
Neoplasia	37 (24.0)	19 (12.6)	0.01
Chemotherapy	6 (3.9)	5 (3.3)	0.784
Menopause, n = 189 (%)*	57 (70.4)	72 (66.7)	0.588
Prostate benign hypertrophy, n = 116 (%)#	48 (64.9)	27 (62.8)	0.822
No risk factor One risk factor Two or more risk factors	7 (4.5)4 (2.6)146 (94.8)	20 (13.2)24 (15.9)106 (70.2)	0.007<0.0001<0.0001

*: Univariate analysis was performed in women. #: Univariate analysis was performed in men.

In multivariate analysis, factors associated with an increased risk of community-onset UTIs due to ESBL-producing *E. coli* included healthcare-associated UTI (odds ratio [OR]: 1.80; 95% confidence interval [CI]: 1.02–3.18; P = 0.041), upper urinary tract infection (OR: 3.05; 95% CI: 1.76–5.29; P < 0.0001), use of antibiotics in the preceding 6 months (OR: 2.28; 95% CI: 1.21–4.30; P = 0.011), and having two or more risk factors (OR: 4.03; 95% CI: 1.73–9.35; P = 0.001) (Table 2). Regarding antibiotic class, previous use of cephalosporin and fluoroquinolones was associated with ESBL-producing *E. coli* (respectively P < 0.001, P = 0.019). There was no correlation between the use of beta-lactam-beta-lactamase inhibitors and ESBL positivity (P = 0.16) (Table 1).

**Table 2:** Multivariate analysis of risk factors for community-onset UTI caused by ESBL-producing Escherichia coli.

	Odds ratio	95% Confidence interval	P-value
Use of antibiotics within previous 6 months	2.28	1.21–4.3	0.011
Healthcare-associated UTI	1.80	1.02–3.18	0.041
Upper UTI	3.05	1.76–5.29	<0.0001
Two or more risk factors	4.03	1.73–9.35	0.001

ESBL-producing *E. coli *isolates had more non-beta-lactam antibiotic resistance than non-ESBL-producing *E. coli *isolates. The resistance rates for ciprofloxacin were 91.6% and 30.5% (P < 0.0001), for TMP-SMX were 65.4% and 35.8% (P < 0.0001), for gentamicin were 64.3% and 7.3% (P < 0.001), for amoxicillin-clavulanate were 99.4% and 23.8% (P < 0.0001), for piperacillin-tazobactam were 19.5% and 4% (P < 0.0001), and for nitrofurantoin were 9.7% and 5.3% (P = 0.193), respectively. Fosfomycin resistance was found only in one ESBL-producing *E. coli *isolate, and there was no resistance to fosfomycin in non-ESBL-producing *E. coli *isolates (Figure).

**Figure:**
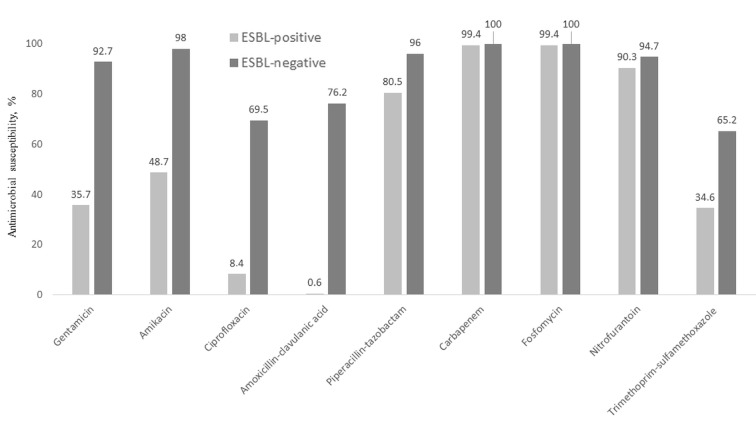
Antimicrobial susceptibility rate (%) of ESBL-positive and -negative Escherichia coli.

## 4. Discussion

ESBL-producing *E. coli* is a precariously increasing pathogen among community-onset UTIs. The ESBL prevalence rate is variable in different geographical regions. For instance, in Norway, it is lower than 2%, but it can also be very high, over 74%, like in Iran [11,12]. ESBL-producing *E. coli* is responsible not only for healthcare-associated but also for community-acquired UTI even in countries with low antibiotic use [5,11,13], and multidrug resistance makes it more difficult to decide the appropriate antibiotic treatment. 

Our results revealed that the ESBL-producing *E. coli* rate was 50.5% in patients with community-onset UTI, and 63.6% of ESBL (+) patients were diagnosed as having healthcare-associated UTIs, while only 29% of ESBL (-) patients had healthcare-associated UTIs. This prevalence seems to be higher than in previous studies [3,14]. In a study from Turkey, the SMART study, the rate of ESBL-positive* E. coli* was much higher in healthcare-associated than community-acquired UTIs during 2011 and 2012, at 50% and 38%, respectively [14]. Another study from Turkey reported a similar ESBL-producing *E. coli* rate in 2006, as 36.7%, among patients with community-acquired UTIs, but ESBL-producing *E. coli* rates were not given for healthcare-associated UTIs [3]. The higher rates of ESBL-producing *E. coli* in community-onset UTIs in our study can be related to higher rates of healthcare-associated UTIs and also a different study period. 

The major independent risk factors for ESBL positivity in our series were the presence of healthcare-associated UTIs, upper urinary tract infection, use of antibiotics in the preceding 6 months, and having two or more risk factors. Healthcare-associated UTIs were associated with an increased risk for ESBL positivity in our analysis. Similarly, previous studies showed that ESBL-producing *E. coli* was more frequent in patients with several healthcare-associated infections including UTIs [15–18]. Indwelling urethral catheter use was the most common reason for healthcare-associated UTI [19,20]. Similarly, the rate of indwelling urethral catheter use was as high as 71.8% in healthcare-associated UTIs in our series.

Our results revealed that there is a threefold increased risk of ESBL-producing *E. coli* in upper UTI cases. We did not come across a study that showed a relationship between upper UTIs and ESBL positivity in our literature review. However, it may be suggested that ESBL-producing *E. coli* is a frequent causative agent in upper UTIs due to a requirement for more frequent hospitalizations, more complications, and the presence of different underlying risk factors in this patient group.

We also found the number of risk factors for ESBL-positive *E. coli* to be important. ESBL positivity was lower in patients with no risk factors or one risk factor, which included being male, old age (≥55 years), use of antibiotics within the previous 6 months, healthcare-associated UTI, upper UTI, frequent UTIs (>3 times/year), renal failure, and neoplasia. In contrast, patients with two or more risk factors were more likely to have ESBL-producing *E. coli*.

When we did not include the presence of two or more risk factors in multivariate analysis, we found that being 55 years of age or older was an independent risk factor. In elderly patients, additional comorbid issues, hospital admission rates, and infectious diseases are more frequent. Antimicrobial drug use, and therefore a greater risk of acquiring antibiotic-resistant bacterial infections, is more frequent among older patients [21]. As in our study, older age and previous use of antibiotics were reported as risk factors for ESBL-positive* E. coli* in previous studies [15,22–25]. In terms of antibiotic class, previous use of cephalosporin and fluoroquinolones was associated with ESBL-producing *E. coli*. Although the use of fluoroquinolone and cephalosporin was reported to be an independent risk factor in many studies [11,18,26,27], the use of fluoroquinolone was not associated with ESBL positivity in some studies [2,25]. 

Extended-spectrum beta-lactamase-producing *E. coli* is not only resistant to beta-lactam antibiotics; there is also a higher rate of coresistance to many antibiotic groups, particularly quinolones, TMP-SMX, and aminoglycosides [24]. Similar to previous reports [12,26,28], our results showed that ESBL-producing *E. coli *isolates had more antibiotic resistance than did non-ESBL-producing *E. coli *isolates. Particularly, amoxicillin-clavulanate, ciprofloxacin, TMP-SMX, and gentamicin resistance was very significant in ESBL-producing *E. coli* isolates. Both ESBL-positive and ESBL-negative *E. coli* isolates remain highly susceptible to carbapenems, fosfomycin, and nitrofurantoin. However, the amoxicillin-clavulanate resistance was higher in the ESBL-positive group than in the studies mentioned above. In a multinational survey, ESBL-producing Enterobacteriaceae isolates from Turkey were more likely to be resistant to amoxicillin-clavulanate than isolates from other regions [23].

A limitation of the present study is the absence of molecular strain typing of ESBL-positive* E. coli* isolates. However, the most prevalent type was CTXM-15 among these isolates, also reported in previous studies from Turkey [3,29]. 

This study revealed that the ESBL rate of *E. coli* isolated from community-onset UTI cases was high in Turkey. The major risk factors for ESBL-producing *E. coli* were the presence of a healthcare-associated UTI, upper urinary tract infection, use of antibiotics in the preceding 6 months, and having two or more risk factors. The increasing prevalence of ESBL-producing *E. coli* makes it difficult to decide the empiric therapy in UTI cases, especially in patients with two or more of the risk factors. Therefore, a better understanding of the epidemiology and risk factors of community-onset UTIs caused by ESBL-producing *E. coli* may have significant implications for the choice of empiric antimicrobial treatment. Considering the risk factors in this patient group can increase treatment success.
